# Multimorbidity: a core priority for learning health systems amidst vertical disease programme cuts

**DOI:** 10.1186/s12961-026-01456-7

**Published:** 2026-02-19

**Authors:** Justin Dixon, Efison Dhodho, Karen Webb, Pugie Chimberengwa, Fionah Mundoga, Kety Choga, Theonevus T. Chinyanga, Nicholas Midzi, Justice Mudavanhu, Lee Nkala, Gwati Gwati, Robert Gongora, Simukai Zizhou, Gerald Shambira, Richard Makurumidze, Stanley M. Midzi, Seye Abimbola, Clare I. R. Chandler, Rashida A. Ferrand

**Affiliations:** 1 The Health Research Unit Zimbabwe, Biomedical Research and Training Institute, Harare, Zimbabwe; 2 Organization for Public Health Interventions and Development, Harare, Zimbabwe; 3 Department of Global Health and Development, London School of Hygiene and Tropical Medicine, 15-17 Tavistock Place, Kings Cross, London, WC1H 9SH UK; 4 National University of Science and Technology, Bulawayo, Zimbabwe; 5 National Institute of Health Research, Ministry of Health and Child Care, Harare, Zimbabwe; 6 Directorate of Non-Communicable Diseases, Ministry of Health and Child Care, Harare, Zimbabwe; 7 Directorate of Policy, Planning, and Health Economics, Ministry of Health and Child Care, Harare, Zimbabwe; 8 Directorate of Health Informatics and Analytics, Ministry of Health and Child Care, Harare, Zimbabwe; 9 Department of Global, Public Health and Family Medicine, Faculty of Medicine and Health Sciences, University of Zimbabwe, Harare, Zimbabwe; 10 World Health Organization, Harare, Zimbabwe; 11 School of Public Health, University of Sydney, Sydney, NSW Australia; 12 Department of Clinical Research, London School of Hygiene and Tropical Medicine, London, UK

**Keywords:** Multimorbidity, Global health, Learning health systems, Zimbabwe

## Abstract

Health systems globally face increasingly complex, multifaceted challenges, cross-cutting many of which is multimorbidity. While rising to multimorbidity has been a slow and incremental process, the recent US funding cuts and rupture of vertical disease programmes may be a pivotal moment for health systems to become at once more integrated, adaptive and self-reliant towards this end. This article considers learning health systems (LHS) as a framework for building such systems, with multimorbidity a core priority and focal point for operationalizing LHS in practice. To illustrate, we draw from an interdisciplinary initiative to catalyse and evaluate a multimorbidity-learning health system in Zimbabwe, centred on three domains of sociotechnical infrastructure: reengineered electronic health records (EHR) to integrate and democratize parallel research, data and decision-support systems; deliberative platforms to support multi-condition sense-making and knowledge translation; and investment in learning sites at service delivery level to facilitate the practical development and iteration of integrated treatment and prevention models. Strategies to build LHS must necessarily be tailored to particular contexts. However, the infrastructural domains and specific mechanisms presented may be valuable for many lower-income countries seeking to emerge from the current funding crisis with the in-house learning capabilities needed to address the complex needs of older, multimorbid populations with less external funding and technical support.

**Supplementary Information:**

The online version contains supplementary material available at 10.1186/s12961-026-01456-7.

## Introduction

As life expectancy increases globally amidst persisting inequities in health and wealth, health systems face increasingly complex, multifaceted challenges, from rapidly rising noncommunicable diseases (NCDs), to new pandemic threats, to climate catastrophe [1]. Crosscutting many of these challenges is rising multimorbidity. Referring to the co-occurrence of two-or-more long-conditions in one person, 37.2% of adults and over half of those aged > 60 years globally experience multimorbidity [2]. In lower-income countries, multimorbidity has been characterized as syndemics of chronic infectious diseases (notably HIV and tuberculosis (TB)) and rapidly rising NCDs, driven by rising life expectancies amidst deepening inequalities, urbanization and environmental degradation [3, 4]. Fast becoming the norm rather than the exception, multimorbidity is among the most pressing emerging health challenges, even called the next global pandemic [5].

The breakdown of the dominant single disease model and urgent need for integrated, adaptive health systems to address multimorbidity and its complexity is increasingly evident. However, how such systems can be realized, especially in lower-resource settings, remains a major challenge. Part of this is an inhibitory global health architecture favouring narrow, vertical responses to single conditions or health challenges, which has contributed to the fragmentation and uneven resourcing of many health systems globally over the past 25 years, including in Africa [6]. A situation analysis in Zimbabwe, for instance, found that its health system, once renowned for its strength, self-reliance and primary care, has been reshaped by vertical programmes across the totality of its functions. This includes the reorientation of health priorities, policies and organizational structures towards the demands of external funders for HIV, TB, malaria and maternal/child heath (at the expense of NCDs and older persons); the proliferation of parallel research, health information and monitoring and evaluation (M&E) systems for these prioritized conditions; and the fragmentation and uneven resourcing of health services, typified by free, decentralized care for HIV and TB contrasting with expensive, hospital-based care for NCDs [7]. The impacts of such shifts were felt most profoundly by people living with multimorbidity. Frequently falling through the gaps in clinical research, guidelines and information systems, they faced uncoordinated facility visits, appointments and queues; multiple prescriptions, dietary, and lifestyle changes; and little information or self-management support – together fuelling spiralling economic and social challenges, complications, further conditions, frailty, disability and decline [7].

Growing recognition of the formidable challenge multimorbidity poses in Zimbabwe and other lower-income countries [8–10] has reinvigorated efforts to integrate services and systems to better respond to the needs of whole persons. However, these have struggled to gain traction. Most remain scaffolded on vertical programmes (for example, integrating NCDs into HIV clinics) [7], restricted to programme and research funded contexts [11, 12] and focused on a narrow range of conditions with less emphasis on patients' social, economic and environmental – syndemic – contexts [6]. The sluggishness of health systems to react to rising multimorbidity and its drivers has brought into the foreground that the way global health has evolved to abstract, universalize and target diseases one-at-a-time has, despite successes in defined areas, worked against the ability of health systems to listen, learn and adapt to increasingly complex health profiles [13]. The recent US funding cuts including the end of the US Agency for International Development (USAID), however, marks a turning point. Overnight, the cuts precipitated a new, uncertain era for global health in which the vertical architectures that sustain the siloed management of single conditions is under unprecedented strain [14]. In this moment of rupture, there is unprecedented opportunity to support and empower health systems to leverage the shifting gravity to meet these emerging challenges head on.

This article considers the potential of the learning health systems (LHS) framework for guiding such a systemic shift. Both paradigm-shifting and pragmatic, the LHS framework reimagines how knowledge, policy and practice can synergize within health systems for more agile, self-reliant responses to complex, cross-cutting challenges such as multimorbidity. We first describe LHS and the value of centring multimorbidity in their formative development. We then describe three domains of infrastructural development to strengthen the learning-enabling environment for multimorbidity, drawing on an initiative to catalyse and evaluate a multimorbidity-learning health system in Zimbabwe. Placing this case into conversation with wider LHS scholarship, we suggest that strategies to develop and institutionalize learning should necessarily be tailored to particular contexts. Yet, the proposed domains may be a useful point of departure for other historically donor-dependent health systems seeking to emerge from the fallout of the current funding crisis with the in-house capabilities needed to address the complex needs of older, multimorbid populations with less external funding and technical support.

### Learning health systems and multimorbidity

Reflecting growing conceptual synergies between health policy and systems research and the social sciences, systems thinking has advanced in recent decades from a mechanistic view of health systems as composites of discrete building blocks – a view that has been argued to perpetuate vertical approaches to health system strengthening [15] – to complex, adaptive, sociotechnical systems encompassing all actors and institutions (including beyond the health sector) that seek to promote, restore and maintain health [16]. Among the latest conceptual iterations of this more dynamic, person-centred focus is the LHS framework. LHS emphasises that while health systems are always adapting, those that invest in capacities and infrastructure to learn from past experience tend to fare better and, as such, should be a key pillar of health system strengthening[17, 18]. Initially centred on clinical care in higher-income settings, scholarship on LHS has more recently been expanded by Sheikh and Abimbola into a more holistic, globally directed framework that encapsulates the generation, acquisition and sharing of knowledge and changing of practices across all (cross-)organizational levels and functions of health systems [18].

**Fig. 1:**
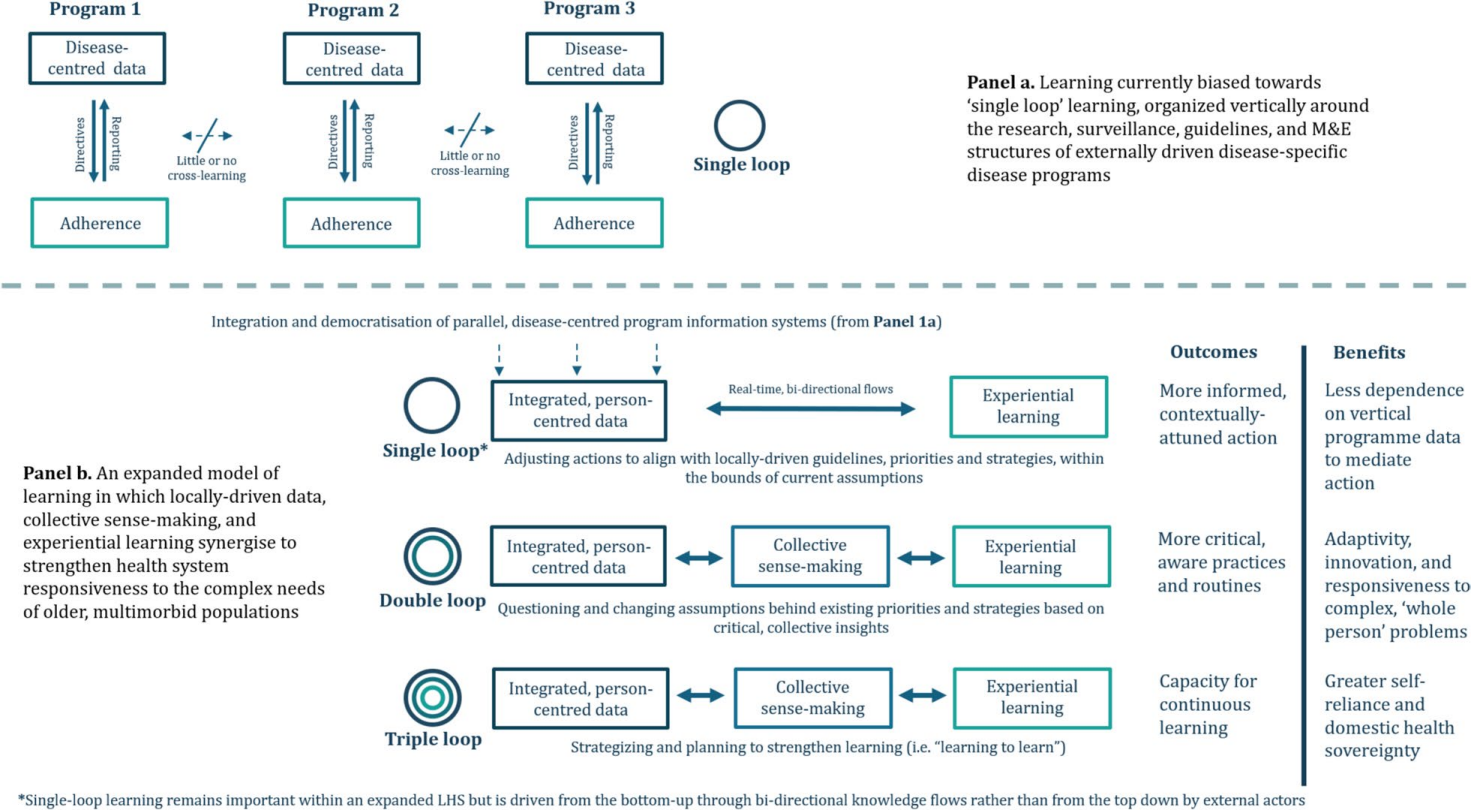
Conceptualizing the means, loops and benefits of learning relative to current health system architectures built around vertical disease programmes. Adapted from [18]

Because of the way global health has evolved, learning within lower-resource health systems tends to be externally driven and focused on the use of information curated by expert analysts to ensure adherence/fidelity to guidelines, protocols and targets, often through monitoring and evaluation (M&E) systems. Referred to as single-loop learning, this can be an important mechanism through which health systems ensure goals are met and functions performed [18]. However, organized vertically around different disease programmes, this has resulted in parallel, reporting-heavy data infrastructures that are more responsive, meaningful and useful to the external actors driving them than those funnelling up information (Fig. 1, a). In LHS, by contrast, learning is driven by domestic actors who are collectively better situated to see the bigger picture, not only to monitor progress against targets but also to critique and, where necessary, change underlying assumptions and translate these into solutions better aligned with local realities and needs (double-loop learning) [18]. Stronger still are systems with longer-term strategies to develop and support continuous learning (triple-loop learning or learning how to learn) [18].

The infrastructural preconditions for such critical, reflective learning cycles include integrating and democratizing information such that it is more accessible, meaningful and useful including to the lowest-level users. Crucially, however, within LHS, learning is more than information and its transfer; it means tapping into a wider repertoire of local knowledge than typically valued in evidence-based global health. This includes the deliberative knowledge gained from bringing together stakeholders across professions, programmes and sectors to better contextualize problems and solutions – which Gilson et al. refer to as collective sensemaking’[19] – and the practical knowledge that comes from trying new things, embracing possible failure and learning from experience [18]. By embedding critical, reflective learning practices across health systems, those working within them can be supported to take greater ownership and sovereignty over domestic knowledge production and its use. Institutionalizing such practices, in turn, contributes to more integrated, adaptive and ultimately more self-reliant health systems (Fig. 1, b) [18].

In a global health context, the bodies of literature around LHS have been weighted towards pandemic preparedness and response, understandably so given the way such shocks necessitate rapid reactivity and reprioritization, as demonstrated during COVID-19 [20]. However, perhaps for this very reason, multimorbidity may be an especially timely focal point for the development of LHS. While multimorbidity may have been argued to be the next global pandemic [5], it requires quite different kinds of learning capacity than the ability to contain and control disease – an orientation that has, in recent times, reinforced vertical approaches and the security agendas of wealthy northern nations [21, 22]. Definitive of the complex, cross-cutting challenges facing health systems today, multimorbidity instead foregrounds the gaps, omissions and unintended consequences of global systems of knowledge, policy and practice built around diseases rather than people, the growing rift between the priorities of health systems and those they serve and the imperative to recentre their realities, needs and social context within the formation of LHS [6, 7]. How an LHS evolves will be shaped or constrained by where it takes root. Thus, a starting point such as multimorbidity, which challenges us to operationalize LHS around the problems of whole persons, may be catalytic for optimizing its transformative potentials while avoiding the familiar contradictions of vertical health system strengthening [6, 7].

## Operationalizing multimorbidity-learning health systems

**Fig. 2:**
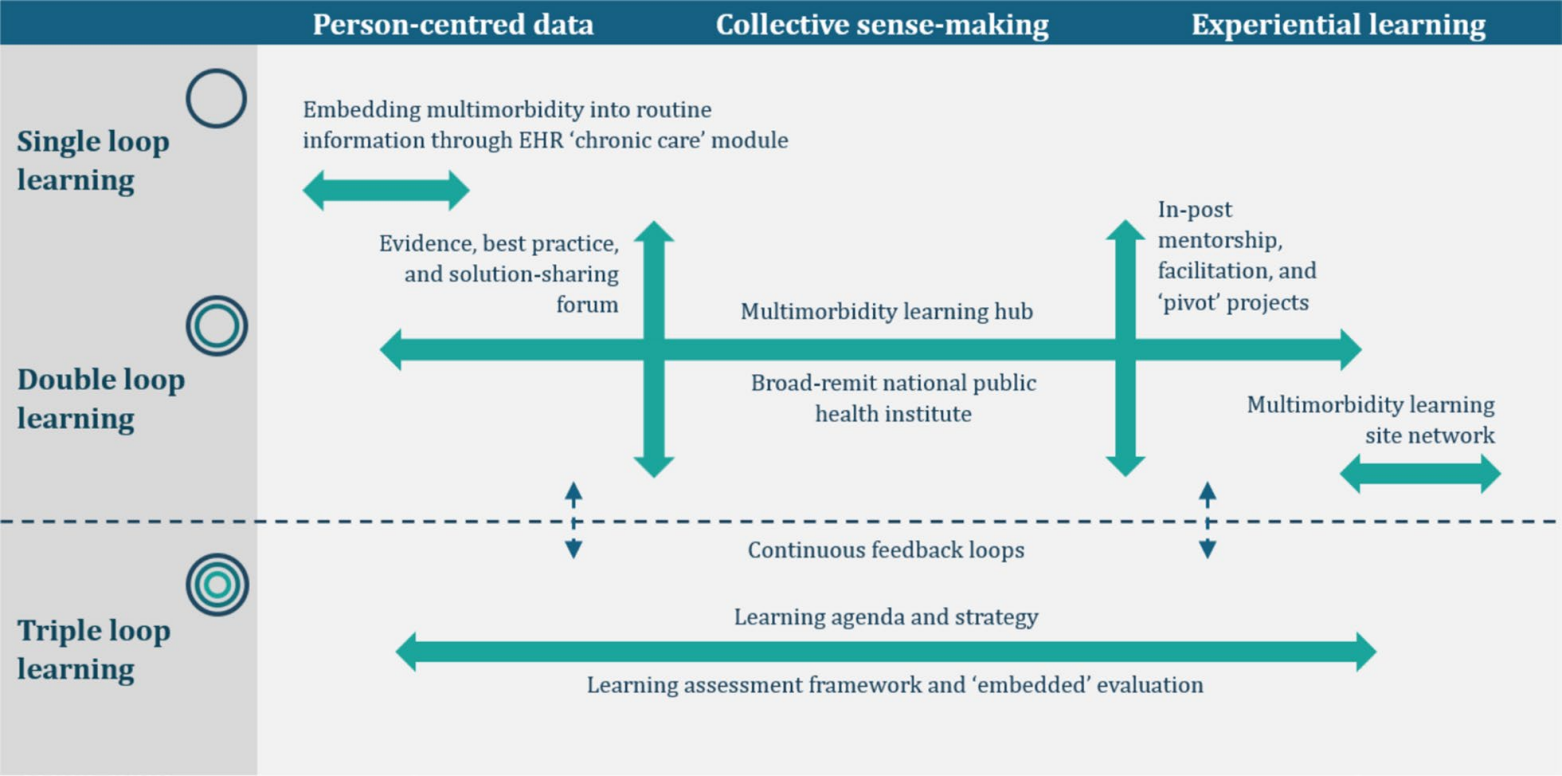
Sociotechnical infrastructure for a multimorbidity-learning health system in Zimbabwe (OptiMuL, 2025–2030). Adapted from [18]

Centring multimorbidity in the operationalization of LHS is deliberately ambitious. It means confronting the deeply entrenched silos, hierarchies and dependencies that shape the social fabric of low-resource health systems and work against the flatter, collaborative ways of working embraced within LHS. But the gravity is shifting. The US funding cuts, for all their harm, have been framed as a long-overdue wakeup call for taking back domestic health sovereignty from externally driven programmes [14], a corollary of which is greater integration and responsiveness to challenges such as multimorbidity that have remained hidden in plain sight [7]. Moreover, with many countries already undergoing health system reform, there are many synergistic initiatives that can be leveraged to support change [18]. There are many possible strategies to strengthen the learning-enabling environment for multimorbidity, whose selection and deployment need to be specific to local agendas and capacities. Yet the similar situations facing many countries present opportunities for shared learning. To advance this conversation, the following outlines three domains of sociotechnical infrastructure of salience to multimorbidity that stem from an interdisciplinary initiative (OptiMuL, 2025–2030) which, on the basis of a situation analysis[7] and national dialogues [23], seeks to catalyse and evaluate a multimorbidity-learning health system in Zimbabwe (Fig. 2). A full project schemata and theory of change is presented as supplementary material (Additional File 1).

### Integrating, strengthening and democratizing health data

With many health systems' learning capacities oriented towards single-loop, information-centred learning, a key infrastructural enabler for multimorbidity-learning is integrating, strengthening and democratizing access to health data. Already, initiatives are underway in population research [24] and chronic disease programmes in several countries [11, 12, 25] to integrate research and routine data systems and make multimorbidity legible in ways that have previously eluded. The LHS framework, however, invites a greater emphasis on ensuring such information is in the service of domestic actors including teams at lower levels of care, such that they are better supported with real-time data to guide action and drive change from below [18, 26]. A range of information-based tools and technologies have been suggested to strengthen bottom-up multimorbidity-learning, including electronic health records (EHR), mobile health (mHealth) apps and AI-powered data collection, analysis and decision-making support systems [27].

In Zimbabwe, we are currently focusing our attention on EHR. As the aforementioned situation analysis showed, Zimbabwe’s health information landscape has been reshaped by parallel research, surveillance and M&E systems for programme-backed diseases, with most of what little is known about NCDs and multimorbidity coming from sporadic nonroutine research and surveys [7]. EHR, introduced in 2016 to replace existing paper-based registers and M&E tools, was presented as a panacea to this scenario by providing a common, harmonized platform across all programmes and conditions. However, notwithstanding the infrastructural, human resource and data security challenges hampering its scale up [28, 29], the system’s initial funding from PEPFAR/CDC meant that it evolved as an assemblage of programme-specific modules (the backbone being HIV care and treatment), a process which has been fragmented, upward-reporting-centred and biased towards specific conditions, with NCDs and multimorbidity continuing to fall through the cracks [7]. However, LHS initiatives in the UK suggest that a functional EHR that is integrated, relevant and accessible in real-time (that is, not as retrospective, often out-of-date dashboards) can be a key learning-enabler, linking decision support, follow-up and longitudinal research and surveillance, while providing feedback loops across health system levels to support decision-making and continuous improvement [30]. Extending these experiences to Zimbabwe’s EHR, we are reengineering a currently underdeveloped chronic care EHR module as a starting point for integrating LHS principles into the EHR ecosystem. Working with front-line health workers, decision-makers and data scientists, the aim is to invert the top-down, disease-driven design orientation of the system’s initial HIV and programme funding by creating an integrated, bottom-up platform to strengthen care, knowledge generation and continuous learning across all common chronic conditions – communicable, NCDs and combinations thereof – managed at primary level (the only level EHR is currently operable). Built by and for those working with the system, the goal is that EHR will become the bedrock of a domestically driven LHS in Zimbabwe and key catalyst for multimorbidity-learning across different health system functions and levels.

### Collectively making sense of multimorbidity for coordinated action

Beyond improving information, dynamic, complexity-attuned decision-making further requires mechanisms that open up available information to collective sense-making among relevant stakeholders [19]. Multimorbidity, as a case exemplar for complexity in health, foregrounds the particular need to build collectives that are able to make sense of challenges that transcend entrenched organizational silos around single diseases and health challenges [6]. One focal point for promoting collective sense-making within deeply fractured health systems such as Zimbabwe’s is national public health institutes (NPHIs), which many nations have (or are planning to establish). While historically oriented towards infectious disease response, NPHIs have the broader remit to strengthen public health capacities, integrate functions across programmes and sectors and foster flatter, more dynamic relations between academia, policy and practice [31]. Zimbabwe’s NPHI, for example (under construction at the time of writing), received catalytic funding to address NCDs and their syndemic drivers as the first priority area around which to build its capacities. NCDs were selected owing to their historic underfunding and the opportunity this presents to reinvigorate the NCD response from an integrated platform that includes not only HIV, TB and other infectious disease threats but also human, animal and environmental sectors within a One Health approach. As such, it is hoped to be a trailblazer among NPHIs in the African region in holistically addressing its emerging NCD and multimorbidity syndemics [3].

With the need for integrated services and systems increasingly evident following the recent funding cuts, a multimorbidity learning hub has been established to strengthen and accelerate learning in this priority area. The learning hub can, after a fashion, be classified as a knowledge translation platform (KTP) [32], an umbrella term for a range of intermediary mechanisms that includes think tanks, policy and systems research/evidence platforms and intelligence units [18], all broadly aiming to bridge the knowing–doing divide to accelerate action to address health challenges. While more commonly set up to address single diseases [33], the learning hub was proposed as a promising disease-neutral focal point for shifting the locus of knowledge-and decision-making from the plethora of externally driven vertical programmes to domestic collectives better positioned to recognize and respond to shifting patterns and syndemic drivers of multimorbidity locally [7, 23]. Led by a cohort of professional fellows [34], the hub brings together academics, decision-makers, healthcare workers and patient representatives to engage in mentored training and development, collective sense-making and action research to catalyse whole-system transformation across several pivot points. EHR and health information, described above, form one such pivot point; others include policy and strategy, public heath medicine, pharmaceutical supply chains and service delivery models. Each pivot point is organized around a fellow-led pivot project, a form of action research to catalyse systems change developed from previous models in Zimbabwe [35]. As such, the hub can be distinguished from other KTP models, for example, think tanks [33], for while its activities include knowledge co-creation – for example, through a regular evidence, best practice and solution-sharing forum – its collaborative activities are more directly action-oriented. KTPs, while translatory of knowledge, still conceptually hold knowing and doing apart; the LHS framework on which the learning hub is premised, by contrast, more deeply transgresses the knowing–doing divide, relocating the locus of knowledge making somewhat further towards the right side of Fig. 2.

### Centring experiential learning in service integration

While pooling available knowledge is crucial for making sense of multimorbidity, LHS ultimately seek to decentralize knowledge making and use to the lowest levels of care and administration [26]. Crucial nodes of knowledge production in this regard are pilot or learning sites, [18] facilities or facility networks that are supported to lead in the identification of promising interventions or innovations for wider scale-up or scale-out, grounded in the experiential and contextual knowledge of front-line implementers, health care teams and patient populations [18]. Of particular valence for multimorbidity is deciding what to do with self-standing HIV clinics/departments and attendant systems, which may now be unsustainable in the wake of the funding cuts. Several countries have issued national directives for district authorities and facilities to immediately integrate chronic care services. Mandating such change is one thing, but in practice, this is hugely complex, with no one-size-fits all solutions. Whether, for instance, to repurpose existing HIV structures into chronic clinics [11], to shift HIV care back into general outpatient departments [7] or to adopt an entirely different approach, may vary by facility type, level of care, resource constraints, demographics, environmental factors and patient and community preference. Here facilities or facility networks that have already achieved a degree of service integration – whether owing to research, technical assistance or independent initiative – can be vital learning sites for determining which organizational arrangements are best fitted to different settings and scenarios and iterating these moving forward.

In Zimbabwe, we are working with two learning sites at primary care level in the country’s metropolitan provinces, Harare and Bulawayo. Both facilities are well-positioned and motivated, having been early implementers of HIV-NCD integration efforts and are optimized sites for EHR – making them especially important in the development and field testing of the chronic care module. During the project, these facilities will be at the forefront of restructuring chronic care services and supportive systems to accommodate a multimorbidity and syndemics perspective. This will be operationalized through several mechanisms including pivot projects [35] led by the learning hub’s fellows and a structured facilitation approach to support the utilization of facility-level EHR data. The latter facilitation approach is based on experience from the UK, which showed that collaboration between academic and health care teams in the form of regular facility visits can support the latter to gain confidence in generating, making sense of and feeding EHR-generated data back into practice in a way that respects the autonomy and self-determination of providers at local level [30, 36]. Although supported by research funding during the project lifecycle, we suggest that this model, centring the expertise of embedded actors all the way down, may represent for a more organic, sustainable and adaptable way of restructuring services than the surgical implementation of rigid, externally-originating chronic care models that predominate in global health.

## Learning to learn with multimorbidity

The above represents one strategy, built around three infrastructural domains, for catalysing a multimorbidity-LHS, centred on the projects, activities and engagements of a learning hub in Zimbabwe. More a catalytic process of systems change than a discrete, transplantable intervention, the intention is not (at this stage) to evidence demonstrable improvements in multimorbidity outcomes – the preoccupation with which has, some have noted, limited and rigidified responses to multimorbidity to date [6]. What we rather seek to illuminate is whether and how, in a historically donor-dependent setting, in-house LHS capabilities can be nurtured to engender a more fundamental systemic responsiveness to rising multimorbidity and the complexity it presents. To the extent that these can be anticipated, the learning outcomes that we therefore hope to observe include new collaborations, connections and confidence to challenge entrenched, externally driven silos and hierarchies organized around single diseases; domestically driven, coproduced knowledge, innovations and collective sense made of multimorbidity in this setting; and perhaps most importantly, further capacities and infrastructure to support continuous learning beyond the project lifecycle (Additional File 1). One possible route for ensuring the sustainability of successes during the project is the longer-term housing or absorption of the learning hub within Zimbabwe’s NPHI, whose broad remit, regional/global networks and commitment to African-led public health [31] make it ideal for more widely applying and institutionalizing LHS practices.

The Zimbabwean initiative is tied to longitudinal research and engagements in this setting [7, 23] and, as such, is not intended to provide a general blueprint of model. However, the similar legacies, epidemiological shifts and funding crises facing many lower-income countries mean that the approach being explored will likely be of much wider relevance in these coming years as the true impacts and implications of the USAID and related funding cuts becomes clearer [14]. To draw out successes, failures and lessons learned both for ourselves and others, we have adopted a case study design informed by the realist research tradition [37], involving a mixed, primarily qualitative series of methods to produce a holistic, longitudinal understanding of learning processes, mechanisms and outcomes within the multimorbidity learning hub. A crucial aspect of the design is that, similar to the dialogues that fed into the initiative [23], most evaluative activities are led by the hub’s collaborators themselves. The aim is not only to continuously feed insights back into the process of learning, increasing chances of attaining shared goals, but in the process to build the triple-loop capacities that figure within its anticipated learning outcomes (Fig. 2). We consider the process one of accompanying learning [19] – neither completely immersed nor completely external, but an embedded mechanism for reflexive, real-time change. Such inbuilt responsiveness is, after all, what LHS is all about: a more closely knit relationship between knowledge and practice within adaptive, aware health systems [18].

## Conclusions

This article presented the challenge and opportunity that multimorbidity presents for LHS and sketched provisional domains and potential mechanisms for realizing multimorbidity-LHS in practice. Strategies to strengthen the learning-enabling environment must, similar to the Zimbabwean initiative on which we have drawn, be painstakingly tailored to context. The horizon of benefit is also medium-to-long term, outstripping the more tangible, immediate wins that tend to draw funding and attention. Yet, if the current moment of rupture in global health funding has made anything clear, it is that counties especially those with few resources can ill afford not to invest in learning [14]. Multimorbidity, as perhaps the most paradigmatic current example of the failures of vertical programming and the urgent need to integrate, domesticize and put people at the centre of services and systems, is an especially appropriate rallying call and focal point for LHS in the current moment, in Zimbabwe no less than anywhere else. With many nations in similar situations, we suggest the material presented may have utility for others seeking to leverage the current funding crisis to build more integrated, adaptive and self-reliant health systems better positioned to embrace complexity and change in this increasingly uncertain era for global health.

## Supplementary Information

Supplementary Material 1. Figure 1: OptiMuL Schemata and provisional theory of change. Description: A figure providing a project schemata and provisional change theory for the case study on which the article draws

## Data Availability

Not applicable.

## Abbreviations

EHR: Electronic health record
LHS: Learning health systems
M&E: Monitoring and evaluation
NPHI: National Public Health Institute
NCD: Noncommunicable disease
TB: Tuberculosis
USAID: US Agency for International Development
KTP: Knowledge translation platform
