# Supplementary material for: Multimorbidity: a core priority for learning health systems amidst vertical disease programme cuts
Source: Health Res Policy Syst. 2026 Feb 19;24:20. doi: 10.1186/s12961-026-01456-7 (PMC12922239; doi:10.1186/s12961-026-01456-7)

Supplementary Figure 1: OptiMuL* Schemata and provisional theory of change

*Multimorbidity and Learning Health Systems: Optimizing Data-to-action (Wellcome Trust ref. 307047/Z/23/Z, 2025-2029)

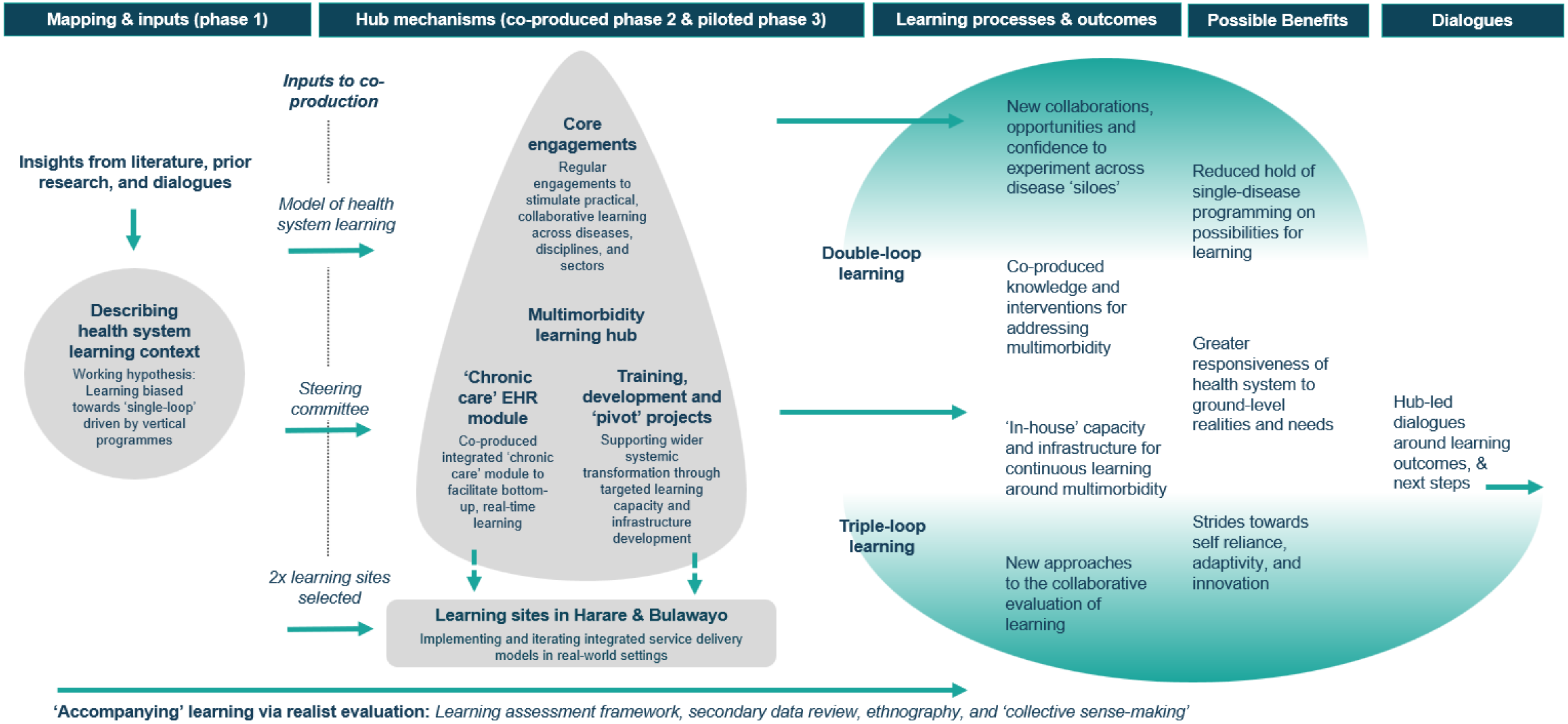

Supplement: Supplementary file 1 — Supplementary Material 1. Figure 1: OptiMuL Schemata and provisional theory of change. Description: A figure providing a project schemata and provisional change theory for the case study on which the article draws [file 12961_2026_1456_MOESM1_ESM.pdf]
